# Minor ginsenoside F1 improves memory in APP/PS1 mice

**DOI:** 10.1186/s13041-019-0495-7

**Published:** 2019-09-05

**Authors:** Junho Han, Jung-Pyo Oh, Miran Yoo, Chang-Hao Cui, Byeong-Min Jeon, Sun-Chang Kim, Jin-Hee Han

**Affiliations:** 1 Department of Biological Sciences, KAIST Institute for the BioCentury, Korea Advanced Institute of Science and Technology, Daejeon, 34141 South Korea; 2 Intelligent Synthetic Biology Center, Korea Advanced Institute of Science and Technology, Daejeon, 34141 South Korea

**Keywords:** Alzheimer’s disease, APP/PS1 mice, Ginsenoside F1, Amyloid-beta plaque, pCREB, BDNF

## Abstract

Ginseng has been shown to produce a cognitive improvement effect. The key molecular components in ginseng that produce pharmacological effects are ginsenosides. Previous studies reported a memory improvement effect of a few major ginsenosides. However, the identity of specific minor ginsenosides mediating such function remains unknown. Here, we report that a minor ginsenoside F1 improves memory function in APPswe/PSEN1dE9 (APP/PS1) double-transgenic Alzheimer’s disease (AD) model mice. After 8-wk oral administration of F1 jelly, we observed that spatial working memory, but not context-dependent fear memory, was restored in AD mice. To search for a possible underlying molecular and cellular mechanism, we investigated the effect of F1 on Aβ plaque. We observed F1 administration reduced the Aβ plaque area and density in the cortex, but not in the hippocampus of AD mice. Next, we tested for the effect of F1 on the expression level of key molecules involved in learning and memory. Results from Western blot assay revealed that an abnormally reduced level of a phosphorylated form of CREB in the hippocampus of AD mice was restored to a normal level by F1 administration. Moreover, in the same animals, BDNF level was augmented in the cortex. Our results, therefore, suggest that minor ginsenoside F1 constitutes a promising target to develop therapeutic agents for AD.

**Electronic supplementary material:**

The online version of this article (10.1186/s13041-019-0495-7) contains supplementary material, which is available to authorized users.

## Introduction

Alzheimer’s disease (AD) is a neurodegenerative disease characterized by a loss of neurons and severe memory impairment. Because AD has become a major social problem globally in an accelerating aging society [1], there has been a dramatic increase in the need for effective treatments that restore or improve memory function. Rescue of the functional deficit is the most important clinical endpoint for patients with approaching the onset of overt dementia [2]. Therefore, identifying a natural compound that is effective to improve memory function in AD is urgently needed.

Ginseng has been utilized as a natural medicinal herb for thousands of years in the East. Recent researches have reported a wide range of therapeutic effects of ginseng, including tumor suppression [3, 4], anti-aging [5], anti-oxidation [6], and cognitive improvement [7–10]. The key molecular components in ginseng that produce such pharmacological effects are ginsenosides. Ginsenosides are natural steroid glycosides, which are abundant in the root of ginseng [11]. Ginsenosides are classified into major and minor ginsenosides, which are produced by the deglycosylation of major ginsenosides [12–14].

After oral administration, major ginsenosides are converted into minor ginsenoside forms by hydrolyzation of the 6- and 20-glucoside bond by intestinal microflora and then absorbed into the body [12]. Non-metabolized major ginsenosides have a low absorption rate in the body and are rapidly eliminated from it [15, 16]. The metabolic rate of intestinal microflora is very low. Moreover, since the composition of intestinal bacteria varies from individual to individual, the pharmacological effects of taking major ginsenosides vary widely from person to person [17]. In contrast, minor ginsenosides are absorbed in the intestine and exert actual pharmacological effects [15]. Therefore, it is critical to identify a single minor ginsenoside that produces a therapeutic effect. However, due to the technical difficulty to obtain a sufficient amount of minor ginsenosides from ginseng for research purposes, most of the extant literature has focused on major ginsenosides for their pharmacological effects. Therefore, a minor ginsenoside with cognitive improvement function in an AD model remains undetermined.

Because major ginsenoside Rg1, the precursor of F1, has been reported to reduce amyloid-beta (Aβ) plaque, modulate neurite outgrowth, and improve cognitive function [18–20], we hypothesized that F1 constitutes a promising candidate. Recently, our research group developed a novel system that enabled mass production of minor ginsenoside F1 from Rg1 [21]. Facilitated by this technical innovation, in the present study, we investigated whether F1 has a therapeutic effect on AD by using AD model mice.

## Results

### Ginsenoside F1 rescues memory impairment in 14-month-old APP/PS1 mice

To test the cognitive improvement effect of F1 in AD, we utilized APPswe/PSEN1dE9 (APP/PS1) double-transgenic AD model mice. The APP/PS1 transgenic mouse expresses chimeric mouse/human amyloid precursor protein (Mo/HuAPP695swe) and a mutant human presenilin 1 (PS1-dE9), which accumulate amyloid beta burden in CNS from 6 to 7 months of age [22–25]. A deficit of learning and memory [26–28] has been reported for these AD model mice.

**Fig. 1:**
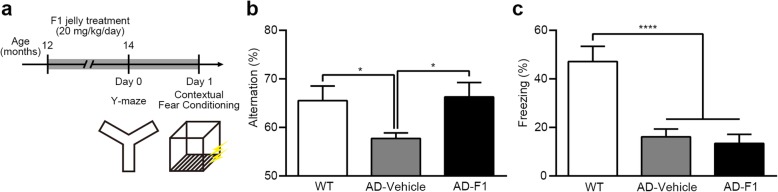
Ginsenoside F1 rescues spatial working memory impairment in APP/PS1 mice. **a** Behavior scheme. Ginsenoside F1 or vehicle jelly was provided for 8 wk. in 12-month-old mice (WT, *n* = 9; AD-Vehicle, *n* = 15; AD-F1, *n* = 10). **b** Y-maze test. Alternative behavior was impaired in AD mice, and ginsenoside F1 rescues impaired alternative behavior (one-way ANOVA, F_(2,31)_ = 5.046, *P* = 0.0127; Tukey post hoc confirmed statistical significance between groups). **c** Contextual fear conditioning. Freezing level was significantly impaired in AD mice during the 24-h contextual fear memory test (one-way ANOVA, F_(2,31)_ = 16.98, *P* < 0.0001; Tukey post hoc confirmed statistical significance between groups), and ginsenoside F1 did not rescue impairment. n values indicate the number of mice. Data are mean ± s.e.m. *P < 0.05, ****P < 0.0001

To assess whether or not F1 can rescue memory impairment in APP/PS1 mice, we conducted a Y-maze test following 8-wk oral administration of F1 jelly (20 mg/kg/day, see Methods) (Fig. 1a). The Y-maze test enables us to test spatial working memory ability, which is mainly dependent on both the hippocampus and cortex region [29–31]. During the Y-maze test, the mice freely moved around the identical three arms, and the spontaneous alternation percentage among the three arms was measured as an index of spatial working memory ability. We separated mice into three groups: F1-treated APP/PS1; vehicle-treated APP/PS1; and wild type littermate control group. The percentage of spontaneous alternation was significantly lower in AD mice compared to that in wild type control mice, indicating memory impairment. Importantly, such a decrease of spontaneous alternation returned to normal level in F1-treated AD mice. This result indicates that F1 improved spatial working memory ability in AD mice (Fig. 1b).

To examine the specificity of the memory improvement effect of F1, we next performed contextual fear conditioning (CFC), which comprised a hippocampus-dependent associative emotional memory [32]. It was reported in AD patients that associated fear-conditioned memory is impaired [33]. The same groups of mice used for the Y-maze test were trained for CFC, and 24-h later tested for long-term memory recall. Consistent with previous reports [34, 35], CFC memory was impaired in APP/PS1 mice. However, the F1 administration did not improve such memory impairment compared to control mice (Fig. 1c). Taken together, our behavioral results suggest that F1 improves working memory function rather than hippocampal-dependent long term memory in AD model mice.

### Ginsenoside F1 reduces Aβ plaques in the cortex of AD mice

**Fig. 2:**
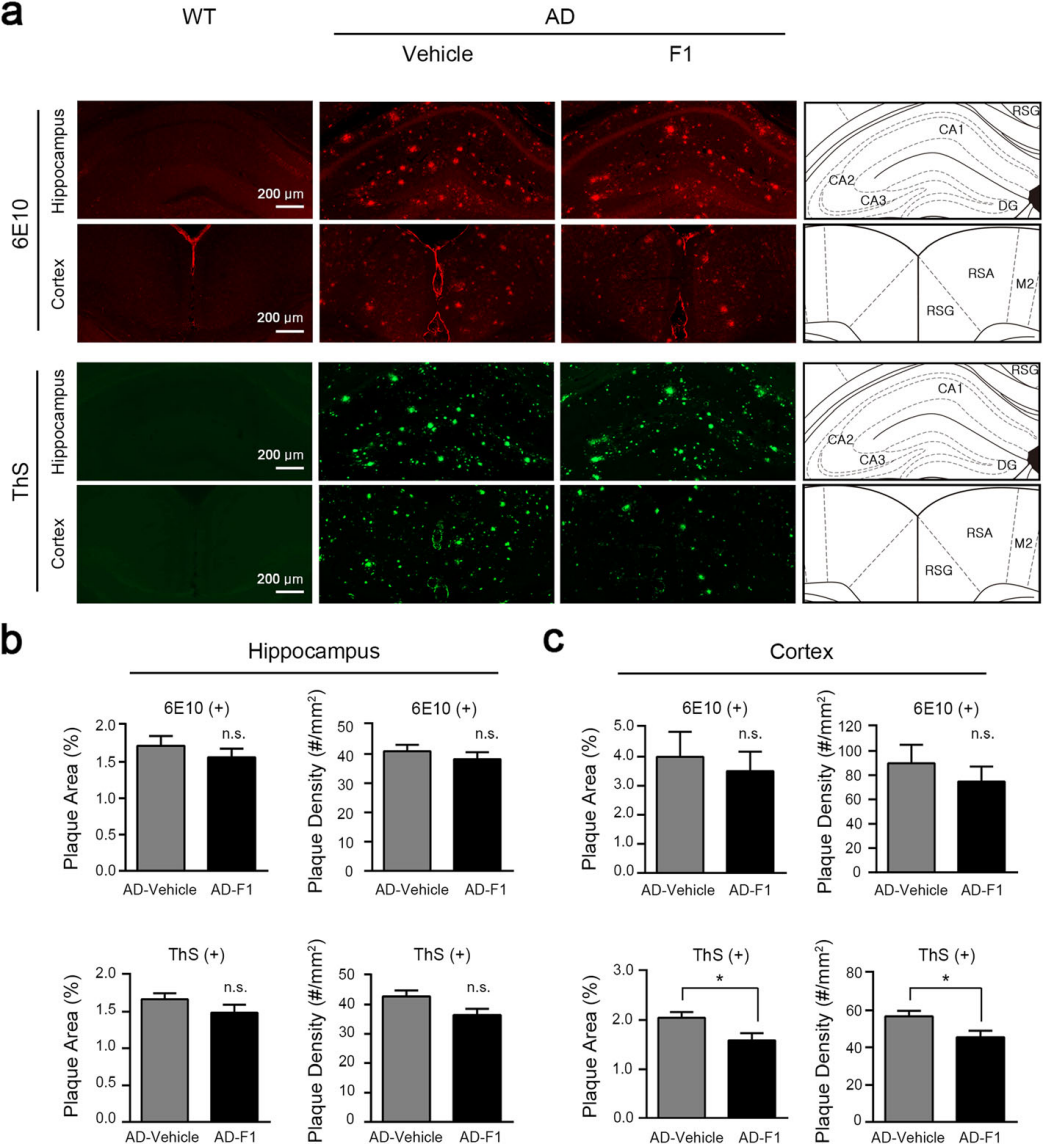
F1 reduces Aβ plaques in the cortex of APP/PS1 mice. 12-month-old APP/PS1 mice were orally administered F1 (20 mg/kg/day; n = 9) or vehicle (*n* = 6) for 8 wk. **a** 6E10-stained Aβ plaques in the hippocampal region (first row) and the retrosplenial cortex region (second row), red; ThS-stained Aβ plaques in the hippocampal region (third row) and the retrosplenail cortex (fourth row), green. **b** In the hippocampal region, the percentage of 6E10 positive plaque area (two-tailed Student’s *t*-test, t_(13)_ = 0.8877, *P* = 0.3908), the density of 6E10 positive plaques (two-tailed Student’s *t*-test, t_(13)_ = 0.7997, *P* = 0.4382), the percentage of ThS positive plaque area (two-tailed Student’s *t*-test, t_(13)_ = 1.217, *P* = 0.2453), and the density of ThS positive plaques (two-tailed Student’s *t*-test, t_(13)_ = 2.065, *P* = 0.0594); (**c**) In the retrosplenial cortex region, the percentage of 6E10 positive plaque area (two-tailed Student’s *t*-test, t_(13)_ = 0.4549, *P* = 0.6567), the density of 6E10 positive plaques (two-tailed Student’s *t*-test, t_(13)_ = 0.7706, *P* = 0.4547), the percentage of ThS positive plaque area (two-tailed Student’s *t*-test, t_(13)_ = 2.238, *P* = 0.04334), and the density of ThS positive plaques (two-tailed Student’s *t*-test, t_(13)_ = 2.298, *P* = 0.0388); The brain schematic diagram [39] shows the brain region of hippocampal and retrosplenial cortex images. n values indicate the number of mice. Data are mean ± s.e.m. *P < 0.05

Aβ deposition in the brain constitutes a key pathophysiological marker of AD [36]. Such Aβ plaque formation has been thought to be the main cause of AD symptoms, including memory deficit, due to its neurotoxic effect [37]. Thus, we reasoned that F1 may improve memory by affecting Aβ plaque. To test this possibility, the plaque counting assay was conducted with 14-month-old APP/PS1 mice. We performed immunostaining with 6E10 antibody and thioflavin S (ThS) staining in the hippocampus and the retrosplenial cortex, which is known as one of the core network brain regions for cognitive functions including episodic memory, navigation, and spatial working memory [38]. 6E10 antibody detects all species of Aβ plaque and amyloid precursor protein, while ThS stains the β-sheeted dense core of Aβ plaque. The area of Aβ burden and the number of Aβ plaque were measured in the hippocampus and cortex region. When we compared the area and density of Aβ plaque in the hippocampal region, no significant difference in both 6E10 positive and ThS positive plaques was found between F1- and vehicle-treated APP/PS1 mice (Fig. 2b). This result indicates no effect of F1 on Aβ plaque in the hippocampus. In the cortex, there was no change in the 6E10 positive plaques by F1 treatment (Fig. 2c), meaning that F1 does not affect the total quantity of Aβ plaques. However, we observed a significant reduction of the ThS positive plaque area and density in the F1 treated AD mice compared to control group (Fig. 2c). Therefore, these results show that ginsenoside F1 inhibits the formation of mature plaques or induces their disaggregation in the cortex, but not in the hippocampus, of AD mice.

### Ginsenoside F1 rescues the expression level of pCREB in the hippocampus and increases the expression level of BDNF in the cortex of APP/PS1 mice

An abnormal decrease of the expression level of the phosphorylated form of CREB (pCREB) [40–43] and BDNF [44–46] has been implicated in a deficit of memory function in AD patients and model mice. Besides, the previous study reported that recovery of cognitive deficit in AD model mice is accompanied by a reduction of Aβ and increase of pCREB and BDNF level [42]. Thus, we investigated whether F1 exerts any effect on the expression level of pCREB and BDNF. We performed Western blot analysis to determine the expression level of pCREB and BDNF in the hippocampus and cortex of 8-month-old APP/PS1 mice following 8 wk. of F1 administration, as done previously.

**Fig. 3:**
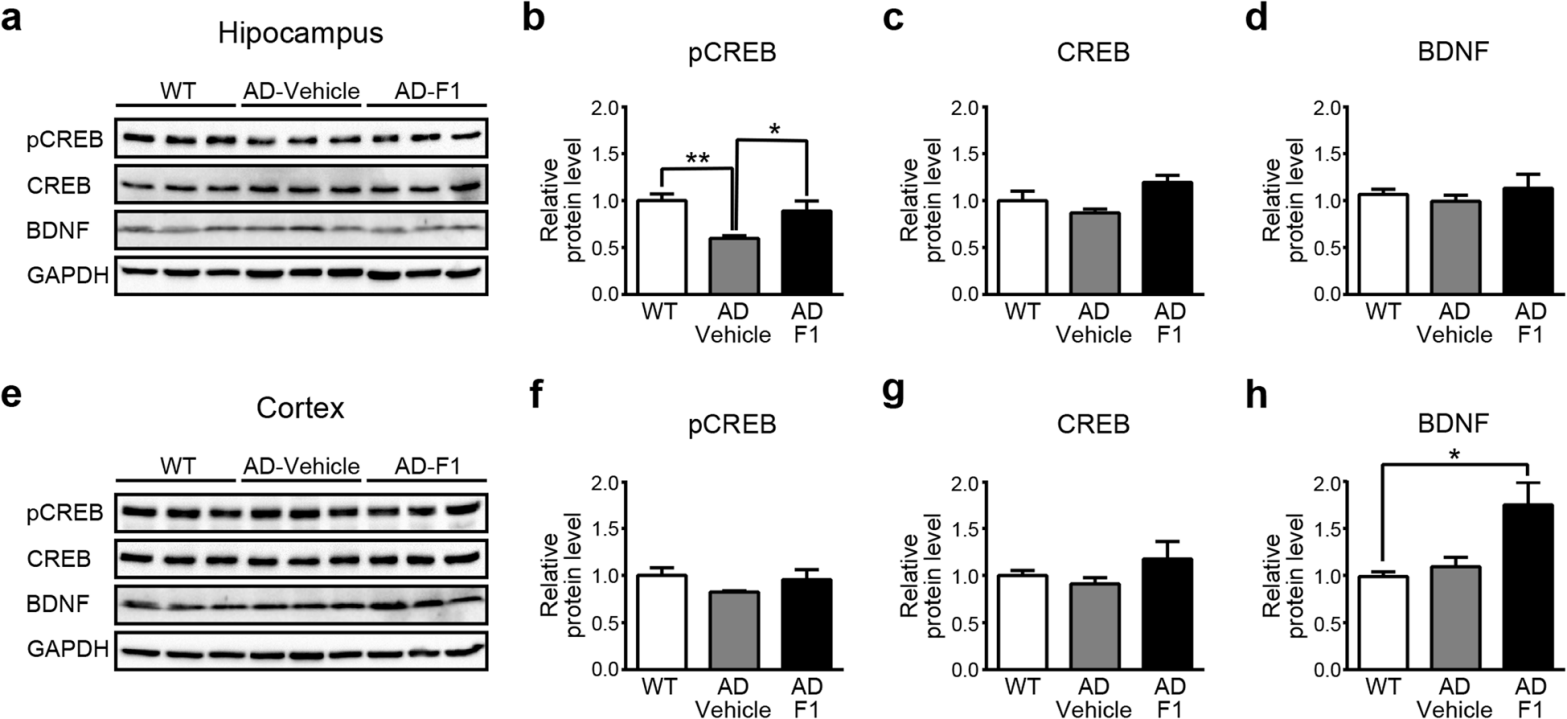
Ginsenoside F1 rescues pCREB and up-regulates BDNF expression level in APP/PS1 mice (a–d) Western blot showing expression levels of pCREB, CREB, and BDNF in the hippocampus. **a** Representative Western blot. **b** pCREB expression in WT mice (WT, *n* = 5), APP/PS1 mice with vehicle administration (AD-Vehicle, *n* = 5), and APP/PS1 mice with F1 administration (AD-F1, *n* = 5) (one-way ANOVA, F_(2,12)_ = 7.623, *P* < 0.01; Tukey post hoc confirmed statistical significance between WT group and AD-Vehicle group, *P* < 0.01; AD-Vehicle group and AD-F1 group, *P* < 0.05). **c** CREB expression levels (WT, *n* = 3; AD-Vehicle, *n* = 3; AD-F1, *n* = 3) (one-way ANOVA, F_(2,6)_ = 4.613, *P* = 0.0612). **d** BDNF expression levels (WT, *n* = 3; AD-Vehicle, *n* = 3; AD-F1, *n* = 3) (one-way ANOVA, F_(2,6)_ = 0.4877, *P* = 0.6364). **e**–**h** Western blot showing expression levels of pCREB, CREB, and BDNF in the cortex. **e** Representative Western blot. **f** pCREB expression levels (WT, *n* = 3; AD-Vehicle, *n* = 3; AD-F1, *n* = 3) (one-way ANOVA, F_(2,6)_ = 1.416, *P* = 0.3136). **g** CREB expression levels (WT, *n* = 3; AD-Vehicle, *n* = 3; AD-F1, *n* = 3) (one-way ANOVA, F_(2,6)_ = 1.273, *P* = 0.3448). **h** BDNF expression levels (WT, *n* = 3; AD-Vehicle, *n* = 3; AD-F1, *n* = 3) (one-way ANOVA, F_(2,6)_ = 7.331, *P* < 0.05; Tukey post hoc confirmed statistical significance between WT group and AD-F1 group, *P* < 0.05). The y-axis indicates normalized protein levels relative to the GAPDH control. n values indicate the number of mice. Data are mean ± s.e.m. *P < 0.05. **P < 0.01. Full-length blots are presented in additional files (See Additional file 1 and Additional file 2)

Consistent with the extant literature [34, 42, 43], we observed that the pCREB level was decreased in the hippocampus of APP/PS1 mice. Such abnormal decrease, however, was restored to the normal level by F1 administration (Fig. 3b). The expression level of total CREB was not significantly changed in all three groups (Fig. 3c), indicating the specific effect of F1 on the pCREB level. Different from pCREB, we found that the expression level of BDNF was not significantly different in the hippocampus of all groups (Fig. 3d). In the cortex, although there were no significant differences in the expression level of pCREB and total CREB among the three groups (Fig. 3f, g), we observed that the BDNF expression increased above normal expression level by F1, as shown in the AD-F1 group (Fig. 3h). Along with the results of Aβ plaque, these results suggest that the recovery of pCREB and the increase of BDNF constitute possible mechanisms of memory improvement by F1 in AD model mice.

## Discussion

In this study, we report for the first time that the administration of minor ginsenoside F1 rescues memory impairment in APP/PS1 double transgenic mice which are known as Alzheimer’s disease model mice. Results from the Y-maze test showed spatial working memory was recovered by F1 in AD mice. To find out the possible underlying mechanism, we examined an effect of F1 on Aβ plaque in the retrosplenial cortex of AD mice. We observed a significant reduction of the ThS positive plaques, but not the 6E10 positive plaques. These results suggest that F1 inhibits the formation of dense Aβ plaques or elicits their disaggregation without changing the total quantity of Aβ plaques in the cortex. Given that the working memory is dependent on the function of the cortex, the reduction of dense Aβ plaques in the cortex may explain the rescue of spatial working memory by F1 in AD mice. In addition to the Aβ plaques, western blot results in this study show F1 increases the level of BDNF above normal levels in the cortex. Previous studies show that the BDNF level is directly correlated with AD severity [47] and the increase of BDNF level is effective to improve cognitive function in AD [48, 49]. Therefore, the increase of BDNF expression in the cortex is also a possible mechanism explaining the improvement of working memory by F1. In the hippocampus, although the number of aggregated forms of Aβ detected with ThS was slightly reduced (*P* = 0.0596), we did not observe any significant effect of F1 on Aβ plaques. Considering the different effect of F1 on Aβ plaques in the cortex and hippocampus, a plausible explanation for the difference of F1 effect on Y-maze versus contextual fear conditioning might be the specific reduction of Aβ plaques only in the cortex. Western blot results showed F1 rescues the pCREB level in the hippocampus to the normal level, but it did not affect the expression level of BDNF. Because contextual fear memory was not improved by F1, it is likely that the restoration of the pCREB level in the hippocampus may not be sufficient to rescue the deficit of hippocampus-dependent memory in AD model mice.

From the western blot result, we failed to see the significant changes of pCREB in the cortex and BDNF in the hippocampus. In our study, western blot analyses were done using 8-months-old mice while behaviors were tested in much older mice. Given the age-dependent progress of AD pathology [50], the relatively young age condition may explain why we failed to see the significant changes of p-CREB in the cortex and BDNF in the hippocampus. Given the mutually positive effects on the expression of BDNF and CREB [51], it is expected that increase of BDNF may in turn cause the same an increase of pCREB level in the cortex after F1 treatment. Similarly, recovery of pCREB level may also lead to an increase of BDNF in the hippocampus. However, these are not what we found. One possible explanation for such discrepancy is that the mutually positive regulation mechanism does not work properly in the AD brain and F1 produces its effect on pCREB and BDNF through other molecular pathways [52–55].

Previous studies reported Rg1, the precursor of F1, rescues cognitive function in AD mice regardless of behavior tasks including Morris water maze, radial arm maze, Y-maze, contextual fear conditioning [7, 19, 56, 57]. Moreover, the Rg1 administration showed a reduction of Aβ plaques in the brain and recovery of memory-related genes like pCREB or BDNF in the hippocampus [19, 56, 57]. In this study, we observed the specific effect of F1 in the Y-maze task which is mainly dependent on the cortex. Consistently, F1 reduced Aβ plaques and increased BDNF expression levels in the cortex but not in the hippocampus. These results suggest that the therapeutic effect of F1 on AD is specific to the cortex, compared to the global effects of Rg1. F1 may have an advantage over Rg1 in terms of the delivery method. In most research, Rg1 was administered via intraperitoneal injection because of the low absorption rate in the intestine of major ginsenosides, a limitation of using Rg1 as a drug or health supplement [15, 16]. However, we show here that F1 can be delivered by oral administration to restore memory impairment with reducing Aβ plaque and increasing pCREB and BDNF expression in AD mice, and thus is a more promising candidate to treat AD.

Considering that synthetic compounds often cause unwanted side effects in many cases, the identification of a natural compound with cognitive improvement function is invaluable to develop medicinal drugs or health supplement foods not only for aged people but also for AD patients. Our results provide evidence that minor ginsenoside F1 improves memory function in AD model mice. Therefore, F1 is a promising target to develop therapeutic agents for memory improvement.

## Materials and methods

### Animals

APPswe/PSEN1dE9 double-transgenic AD mice with a B6 × C3 background and B6 × C3 wild type mice were purchased from the Jackson Laboratory (MMRRC Stock No. 034829-JAX). Heterozygous males were bred with wild type females. Offspring were genotyped by using a standard PCR protocol detecting PSEN1 transgene. Mice that did not express the transgene were used as wild type controls. Mice were housed on a 12-h light/dark cycle at a constant temperature (21–23 °C) and humidity (40–60%). Food and water were available ad libitum. All procedures and protocols were approved by the Animal Ethics Committee at the Korea Advanced Institute of Science and Technology. All experiments were performed in accordance with the guideline of Institutional Animal Care and Use Committee.

### Preparation of ginsenoside F1

Ginsenoside F1 (> 95% pure) was prepared using an enzymatic method from *Panax ginseng* extract as previously reported [21] and isolated using Recycling Preparative HPLC (Japan Analytical Industry Co., Ltd.) with JAIGEL-ODS-AP column (10 mm, 500 × 20 mm id, Japan Analytical Industry Co., Ltd.).

### F1 treatment

To test the effect of F1 on AD, F1 was orally administrated via gelatin-based jelly at a dose level of 20 mg/kg/day. Gelatin-based jelly was prepared as previously described [58, 59]. For a 1-d dose of jelly, 0.6 mg of ginsenoside F1 was dissolved in 0.45 ml of 20% Splenda solution. F1 solution was further mixed with 1.35 ml of 14% gelatin, 20% Splenda solution, and 0.15 ml chocolate-flavoring in a 24-well plate. A piece of jelly (~ 1.9 mg) was provided, and complete intake of jelly was confirmed daily.

Mice were 12-months-old when F1 treatment began, and all the mice were male for behavioral tests and immunohistochemistry of amyloid beta plaque. AD mice and wild type mice were separated into three groups: F1-treated AD; vehicle-treated AD; and non-treated wild type mice. After 8-wk administration of F1, behavioral tests and immunohistochemistry test were performed. F1 was administered to six-month-old male and female mice for 8 wk. for Western blot test.

### Y-maze

The Y-maze test was performed after 8-wk oral administration of F1. Mice were handled for 5 min on 3 d prior to the behavioral experiments. The apparatus has three identical arms (30 cm long, 5 cm wide, and 12 cm high walls) that converge to the center with 120° angles from each other. At the beginning of the test, the mice were placed at one end of an arm and allowed to move freely for 8 min. After the behavioral experiment, mice were returned back to their home cage. All behavioral procedures were recorded by a video camera, and the sequence of entry was manually counted. Entry was counted when all four paws of the mice were in the arm. The percent of alternation was calculated as the number of three consecutive different arm entries over the total number of entries minus two:

$$ \mathrm{Alternation}\ \left(\%\right)=\frac{number\ of\ alternation}{total\ number\ of\ entry-2}\times 100. $$

### Contextual fear conditioning

For contextual fear conditioning (CFC), mice were handled for 5 min on 3 d prior to conditioning. On conditioning day, mice were placed in a fear conditioning chamber (Coulbourn Instruments) with a metal grid floor. Mice were allowed to explore the context for 150 s, and 2 s of 0.5 mA electrical foot shock was delivered twice (120 s inter-stimulus-interval). Mice were left in the conditioning chamber for an additional 30 s and placed back in their home cage. For the contextual fear memory test, mice were placed back into the same context 24 h after conditioning. Behavior of mice was recorded for 5 min, and mice were returned to their home cage. Freezing was automatically scored using FreezeFrame3.0 software (Coulbourn Instruments).

### Brain sample preparation

Mice were anesthetized with 2.5% avertin by intraperitoneal injection. Mice were perfused with phosphate-buffered saline (PBS) and then fixed with cold 4% paraformaldehyde (PFA). After perfusion, brain samples were stored in 4% PFA overnight for post-fixation. Fixed brain samples were immersed in 30% sucrose in filtered PBS until they sank to the bottom of the vial at 4 °C for dehydration. Dehydrated brains were fixed on a disk with OCT compound at − 20 °C. 40-μm thickness sections of hippocampal tissue were collected using Cryostat (Leica CM1850, Leica Biosystems).

### Immunohistochemistry and thioflavin S staining

To visualize amyloid beta plaque in brain sections, amyloid beta was stained by 6E10 and thioflavin S. After three times of PBS washing, brain sections were blocked with blocking solution (0.1% BSA, 0.2% Triton X-100, 2% goat serum in PBS). Brain sections were then incubated with rabbit anti-6E10 monoclonal antibody (BioLegend, 803,015, 1:2000) overnight at room temperature. Next, Alexa fluor-594 conjugated goat anti-rabbit antibody (Molecular Probes, A-11037, 1:1000) was used as a secondary antibody. For thioflavin S staining, brain sections were incubated for 10 min in 0.0008% ThS dissolved in 50% ethanol. Sections were washed with 50% ethanol and PBS twice each. The sections were mounted with VECTASHIELD Antifade Mounting Media with DAPI (H-1200-10, Vector Laboratories) on glass slides. Images were taken on a slide scanner (ZEISS Axio Scan.Z1, Carl ZEISS). To analyze the number of amyloid beta plaque and plaque area, the hippocampus region and the retrosplenial cortex region of coronal brain sections (bregma − 1.6 to − 2.4 mm) was analyzed by using the Image-J program (NIH). Plaques less than 10 μm in diameter were not scored.

### Western blot

To test the effect of F1 on the expression level of pCREB and BDNF, 8-month-old AD mice and age-matched wild type mice were used: F1-treated AD (*n* = 3); vehicle-treated AD (n = 3); and age-matched non-treated wild type mice (*n* = 5). Mice were anesthetized with isoflurane, and brains were extracted. Hippocampus tissue and whole cortex tissue were collected by dissecting brain (bregma − 1 to − 3 mm), and lysed in 100 μl of ice-cold lysis buffer (50 mM HEPES pH 8.0, 150 mM NaCl, 10% glycerol, 1% Triton X-100, 12 mM MgCl_2_, 20 mM EGTA, 10 mM NaPPi, 100 mM NaF, 10 mM Na-Orthovanadate, 1 mM DTT) containing a protease inhibitor cocktail (Roche, 11,836,153,001). Total protein concentrations were measured by Bradford assay. Because CREB and pCREB have almost the same protein size, they had to be blotted in separate gels. Two identical protein samples from the same tissue lysate were prepared and processed in parallel. Proteins (30 μg per lane) were resolved by SDS-PAGE, and transferred to PVDF membranes by using the Trans-Blot Turbo Blotting System (Bio-Rad). Membranes were blocked by 5% NFDM (nonfat dried milk) in TNTX buffer (50 mM Tris-HCl, pH 7.5, 200 mM NaCl, 0.2% Triton X-100) for 30 min at room temperature. We used 3% BSA instead of 5% NFDM for blocking membrane that was used to blot pCREB. After blocking, membranes were incubated with primary antibodies (anti-CREB antibody, Cell Signaling, 9197S, 1:1000; anti-phospho-CREB (Ser133) antibody, Millipore, 06–519, 1:2000; anti-BDNF antibody, Abcam, ab226843, 1:2000) in 3% BSA overnight at 4 °C. HRP-conjugated goat anti-rabbit IgG (Millipore, 12–348, 1:2000) was used as a secondary antibody. Signals were developed with ECL solution (GE Healthcare, RPN2232) and detected using ChemiDoc MP imaging system (Bio-Rad). The results were analyzed using ImageLab software (Bio-Rad). BDNF and GAPDH were stained on the same membrane after stripping the CREB or pCREB antibodies using Restore Western Blot Stripping Buffer (21,059, Thermo Fisher Scientific). As a loading control, anti-GAPDH antibody (Invitrogen, MA5–15738, 1:2000) was used. All Western blot data were normalized to GAPDH expression level for comparison.

### Statistical analysis

GraphPad Prism 6 was utilized to obtain graphs and perform statistical analysis. To confirm normality of data, we conducted D’Agostino-Pearson or Shapiro-Wilk test. A two-tailed, unpaired student’s *t*-test was performed to analyze plaque density and area. One-way ANOVA followed by Tukey’s post-hoc was conducted to analyze the behavioral task and Western blot assay data. Error bars represent the s.e.m.

## Additional files

Additional file 1: **Figure S1.** Uncropped western blot images for hippocampus samples related with Fig. 3a. (PDF 126 kb)

Additional file 2: **Figure S2.** Uncropped western blot images for cortex samples related with Fig. 3e. (PDF 132 kb)

## Data Availability

The datasets generated and analyzed during the current study are available from the corresponding author upon reasonable request.

## Abbreviations

AD: Alzheimer’s disease
APP/PS1: APPswe/PSEN1dE9
Aβ: Amyloid beta
BDNF: Brain-derived neurotrophic factor
CFC: Contextual fear conditioning
CREB: cAMP response element-binding protein
Mo/HuAPP695swe: Chimeric mouse/ human amyloid precursor protein
PBS: Phosphate-buffered saline
pCREB: Phosphorylated form of CREB
PFA: Paraformaldehyde
PS1-dE9: Mutant human presenilin 1
ThS: Thioflavin S

## References

[1] Alzheimer’s Association (2018). 2018 Alzheimer’s disease facts and figures. Alzheimers Dement.

[2] US Food and Drug Administration, Center for Drug Evaluation and Research (CDER), Center for Biologics Evaluation and Research (CBER). Early Alzheimer’s disease: developing drugs for treatment–guidance for industry 2018. https://www.fda.gov/downloads/Drugs/GuidanceComplianceRegulatoryInformation/Guidances/UCM596728.pdf. Accessed Feb 2018.

[3] Yang L, Xin J, Zhang Z, Yan H, Wang J, Sun E (2016). TPGS-modified liposomes for the delivery of ginsenoside compound K against non-small cell lung cancer: formulation design and its evaluation in vitro and in vivo. J Pharm Pharmacol.

[4] Mochizuki M, Yoo Y, Matsuzawa K, Sato K, Saiki I, Tonooka S (1995). Inhibitory effect of tumor metastasis in mice by saponins, ginsenoside-Rb2, 20 (R)-and 20 (S)-ginsenoside-Rg3, of red ginseng. Biol Pharm Bull.

[5] Cheng Y, SHEN LH, ZHANG JT (2005). Anti-amnestic and anti-aging effects of ginsenoside Rg1 and Rb1 and its mechanism of action. Acta Pharmacol Sin.

[6] Cho WC, Chung WS, Lee SK, Leung AW, Cheng CH, Yue KK (2006). Ginsenoside re of Panax ginseng possesses significant antioxidant and antihyperlipidemic efficacies in streptozotocin-induced diabetic rats. Eur J Pharmacol.

[7] Quan Q, Wang J, Li X, Wang Y (2013). Ginsenoside Rg1 decreases Aβ1–42 level by upregulating PPARγ and IDE expression in the hippocampus of a rat model of Alzheimer's disease. PLoS One.

[8] Lee ST, Chu K, Sim JY, Heo JH, Kim M (2008). Panax ginseng enhances cognitive performance in Alzheimer disease. Alzheimer Dis Assoc Disord.

[9] Kim HJ, Jung SW, Kim SY, Cho IH, Kim HC, Rhim H (2018). Panax ginseng as an adjuvant treatment for Alzheimer's disease. Journal of ginseng research.

[10] Smith I, Williamson EM, Putnam S, Farrimond J, Whalley BJ (2014). Effects and mechanisms of ginseng and ginsenosides on cognition. Nutr Rev.

[11] Attele AS, Wu JA, Yuan CS (1999). Ginseng pharmacology: multiple constituents and multiple actions. Biochem Pharmacol.

[12] Tawab MA, Bahr U, Karas M, Wurglics M, Schubert-Zsilavecz M (2003). Degradation of ginsenosides in humans after oral administration. Drug Metab Dispos.

[13] Hasegawa H (2004). Proof of the mysterious efficacy of ginseng: basic and clinical trials: metabolic activation of ginsenoside: deglycosylation by intestinal bacteria and esterification with fatty acid. J Pharmacol Sci.

[14] Ruan JQ, Leong WI, Yan R, Wang YT (2010). Characterization of metabolism and in vitro permeability study of notoginsenoside R1 from Radix notoginseng. J Agric Food Chem.

[15] Feng L, Wang L, Hu C, Jiang X (2010). Pharmacokinetics, tissue distribution, metabolism, and excretion of ginsenoside Rg 1 in rats. Arch Pharm Res.

[16] Liu H, Yang J, Du F, Gao X, Ma X, Huang Y (2009). Absorption and disposition of ginsenosides after oral administration of Panax notoginseng extract to rats. Drug Metab Dispos.

[17] Bae EA, Shin JE, Kim DH (2005). Metabolism of ginsenoside re by human intestinal microflora and its estrogenic effect. Biol Pharm Bull.

[18] Mook-Jung I, Hong HS, Boo JH, Lee KH, Yun SH, Cheong MY (2001). Ginsenoside Rb1 and Rg1 improve spatial learning and increase hippocampal synaptophysin level in mice. J Neurosci Res.

[19] Fang F, Chen X, Huang T, Lue LF, Luddy JS, Yan SS (2012). Multi-faced neuroprotective effects of Ginsenoside Rg1 in an Alzheimer mouse model. Biochimica et Biophysica Acta (BBA)-Molecular Basis of Disease.

[20] Li N, Zhou L, Li W, Liu Y, Wang J, He P (2015). Protective effects of ginsenosides Rg1 and Rb1 on an Alzheimer's disease mouse model: a metabolomics study. J Chromatogr.

[21] Kim JK, Cui CH, Yoon MH, Kim SC, Im WT (2012). Bioconversion of major ginsenosides Rg1 to minor ginsenoside F1 using novel recombinant ginsenoside hydrolyzing glycosidase cloned from Sanguibacter keddieii and enzyme characterization. J Biotechnol.

[22] Jankowsky JL, Slunt HH, Gonzales V, Jenkins NA, Copeland NG, Borchelt DR (2004). APP processing and amyloid deposition in mice haplo-insufficient for presenilin 1. Neurobiol Aging.

[23] Jankowsky JL, Fadale DJ, Anderson J, Xu GM, Gonzales V, Jenkins NA (2003). Mutant presenilins specifically elevate the levels of the 42 residue β-amyloid peptide in vivo: evidence for augmentation of a 42-specific γ secretase. Hum Mol Genet.

[24] Ordonez-Gutierrez L, Fernandez-Perez I, Herrera JL, Anton M, Benito-Cuesta I, Wandosell F (2016). AβPP/PS1 transgenic mice show sex differences in the cerebellum associated with aging. J Alzheimers Dis.

[25] Ordóñez-Gutiérrez L, Antón M, Wandosell F (2015). Peripheral amyloid levels present gender differences associated with aging in AβPP/PS1 mice. J Alzheimers Dis.

[26] Reiserer RS, Harrison FE, Syverud DC, McDonald MP (2007). Impaired spatial learning in the APPSwe+ PSEN1ΔE9 bigenic mouse model of Alzheimer’s disease. Genes Brain Behav.

[27] Kim HY, Kim HV, Yoon JH, Kang BR, Cho SM, Lee S (2014). Taurine in drinking water recovers learning and memory in the adult APP/PS1 mouse model of Alzheimer's disease. Sci Rep.

[28] Savonenko A, Xu GM, Melnikova T, Morton JL, Gonzales V, Wong MP (2005). Episodic-like memory deficits in the APPswe/PS1dE9 mouse model of Alzheimer's disease: relationships to β-amyloid deposition and neurotransmitter abnormalities. Neurobiol Dis.

[29] Lalonde R (2002). The neurobiological basis of spontaneous alternation. Neurosci Biobehav Rev.

[30] Divac I, Wikmark RGE, Gade A (1975). Spontaneous alternation in rats with lesions in the frontal lobes: an extension of the frontal lobe syndrome. Physiol Psychol.

[31] Delatour B, Gisquet-Verrier P (1996). Prelimbic cortex specific lesions disrupt delayed-variable response tasks in the rat. Behav Neurosci.

[32] Phillips R, LeDoux JE (1992). Differential contribution of amygdala and hippocampus to cued and contextual fear conditioning. Behav Neurosci.

[33] Hamann S, Monarch ES, Goldstein FC (2002). Impaired fear conditioning in Alzheimer’s disease. Neuropsychologia..

[34] Kim HY, Kim HV, Jo S, Lee CJ, Choi SY, Kim DJ, Kim Y (2015). EPPS rescues hippocampus-dependent cognitive deficits in APP/PS1 mice by disaggregation of amyloid-β oligomers and plaques. Nat Commun.

[35] Cramer PE, Cirrito JR, Wesson DW, Lee CD, Karlo JC, Zinn AE (2012). ApoE-directed therapeutics rapidly clear β amyloid and reverse deficits in AD mouse models. Science..

[36] Masters CL, Selkoe DJ (2012). Biochemistry of amyloid β-protein and amyloid deposits in Alzheimer disease. Cold Spring Harbor perspectives in medicine.

[37] Yankner BA, Dawes LR, Fisher S, Villa-Komaroff L, Oster-Granite ML, Neve RL (1989). Neurotoxicity of a fragment of the amyloid precursor associated with Alzheimer's disease. Science..

[38] Vann SD, Aggleton JP, Maguire EA (2009). What does the retrosplenial cortex do?. Nat Rev Neurosci.

[39] Paxinos G, Franklin BJ. The Mouse Brain in Stereotaxic Coordinates. 2nd ed. Academic Press;2001.

[40] Pugazhenthi S, Wang M, Pham S, Sze CI, Eckman CB (2011). Downregulation of CREB expression in Alzheimer's brain and in Aβ-treated rat hippocampal neurons. Mol Neurodegener.

[41] Bartolotti N, Bennett DA, Lazarov O (2016). Reduced pCREB in Alzheimer’s disease prefrontal cortex is reflected in peripheral blood mononuclear cells. Mol Psychiatry.

[42] Hou Y, Aboukhatwa MA, Lei DL, Manaye K, Khan I, Luo Y (2010). Anti-depressant natural flavonols modulate BDNF and beta amyloid in neurons and hippocampus of double TgAD mice. Neuropharmacology..

[43] Zhang J, Guo J, Zhao X, Chen Z, Wang G, Liu A (2013). Phosphodiesterase-5 inhibitor sildenafil prevents neuroinflammation, lowers beta-amyloid levels and improves cognitive performance in APP/PS1 transgenic mice. Behav Brain Res.

[44] Ke HC, Huang HJ, Liang KC, Hsieh-Li HM (2011). Selective improvement of cognitive function in adult and aged APP/PS1 transgenic mice by continuous non-shock treadmill exercise. Brain Res.

[45] Caccamo A, Maldonado MA, Bokov AF, Majumder S, Oddo S (2010). CBP gene transfer increases BDNF levels and ameliorates learning and memory deficits in a mouse model of Alzheimer's disease. Proc Natl Acad Sci U S A.

[46] Phillips HS, Hains JM, Armanini M, Laramee GR, Johnson SA, Winslow JW (1991). BDNF mRNA is decreased in the hippocampus of individuals with Alzheimer's disease. Neuron..

[47] Peng S, Wuu J, Mufson EJ, Fahnestock M (2005). Precursor form of brain-derived neurotrophic factor and mature brain-derived neurotrophic factor are decreased in the pre-clinical stages of Alzheimer's disease. J Neurochem.

[48] Blurton-Jones M, Kitazawa M, Martinez-Coria H, Castello NA, Müller FJ, Loring JF (2009). Neural stem cells improve cognition via BDNF in a transgenic model of Alzheimer disease. Proc Natl Acad Sci U S A.

[49] Nagahara AH, Merrill DA, Coppola G, Tsukada S, Schroeder BE, Shaked GM (2009). Neuroprotective effects of brain-derived neurotrophic factor in rodent and primate models of Alzheimer's disease. Nat Med.

[50] Hu YS, Long N, Pigino G, Brady ST, Lazarov O (2013). Molecular mechanisms of environmental enrichment: impairments in Akt/GSK3β, neurotrophin-3 and CREB signaling. PLoS One.

[51] Tao X, Finkbeiner S, Arnold DB, Shaywitz AJ, Greenberg ME (1998). Ca2+ influx regulates BDNF transcription by a CREB family transcription factor-dependent mechanism. Neuron..

[52] Saura CA, Valero J (2011). The role of CREB signaling in Alzheimer’s disease and other cognitive disorders. Rev Neurosci.

[53] McDowell KA, Hutchinson AN, Wong-Goodrich SJ, Presby MM, Su D, Rodriguiz RM (2010). Reduced cortical BDNF expression and aberrant memory in Carf knock-out mice. J Neurosci.

[54] Timmusk T, Palm K, Metsis M, Reintam T, Paalme V, Saarma M, Persson H (1993). Multiple promoters direct tissue-specific expression of the rat BDNF gene. Neuron..

[55] Chen WG, West AE, Tao X, Corfas G, Szentirmay MN, Sawadogo M (2003). Upstream stimulatory factors are mediators of Ca2+−responsive transcription in neurons. J Neurosci.

[56] Li F, Wu X, Li J, Niu Q (2016). Ginsenoside Rg1 ameliorates hippocampal long-term potentiation and memory in an Alzheimer's disease model. Mol Med Rep.

[57] Shi YQ, Huang TW, Chen LM, Pan XD, Zhang J, Zhu YG, Chen XC (2010). Ginsenoside Rg1 attenuates amyloid-β content, regulates PKA/CREB activity, and improves cognitive performance in SAMP8 mice. J Alzheimers Dis.

[58] Zhang L, Lee NJ, Nguyen AD, Enriquez RF, Riepler SJ, Stehrer B (2010). Additive actions of the cannabinoid and neuropeptide Y systems on adiposity and lipid oxidation. Diabetes Obes Metab.

[59] Zhang L. Voluntary oral administration of drugs in mice. Protocol Exchange. 2011;10.

