# Supplementary figures and images for: Minor ginsenoside F1 improves memory in APP/PS1 mice
Source: Mol Brain. 2019 Sep 5;12:77. doi: 10.1186/s13041-019-0495-7 (PMC6728944; doi:10.1186/s13041-019-0495-7)

**Additional Fig. 1** Uncropped western blot images for hippocampus samples (related with Fig 3a)

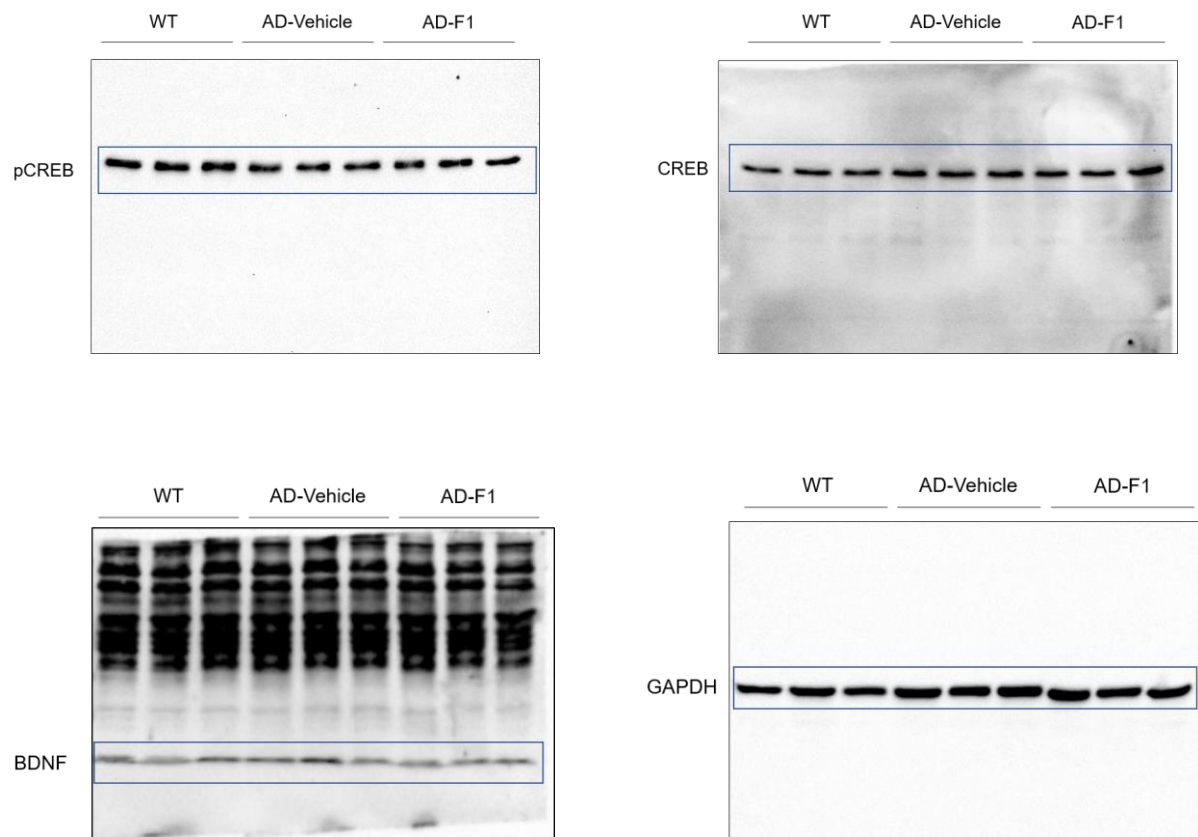

Supplement: Supplementary file 1 — Figure S1. Uncropped western blot images for hippocampus samples related with Fig. 3a. (PDF 126 kb) [file 13041_2019_495_MOESM1_ESM.pdf]

**Additional Fig. 2** Uncropped western blot images for cortex samples (related with Fig 3e)

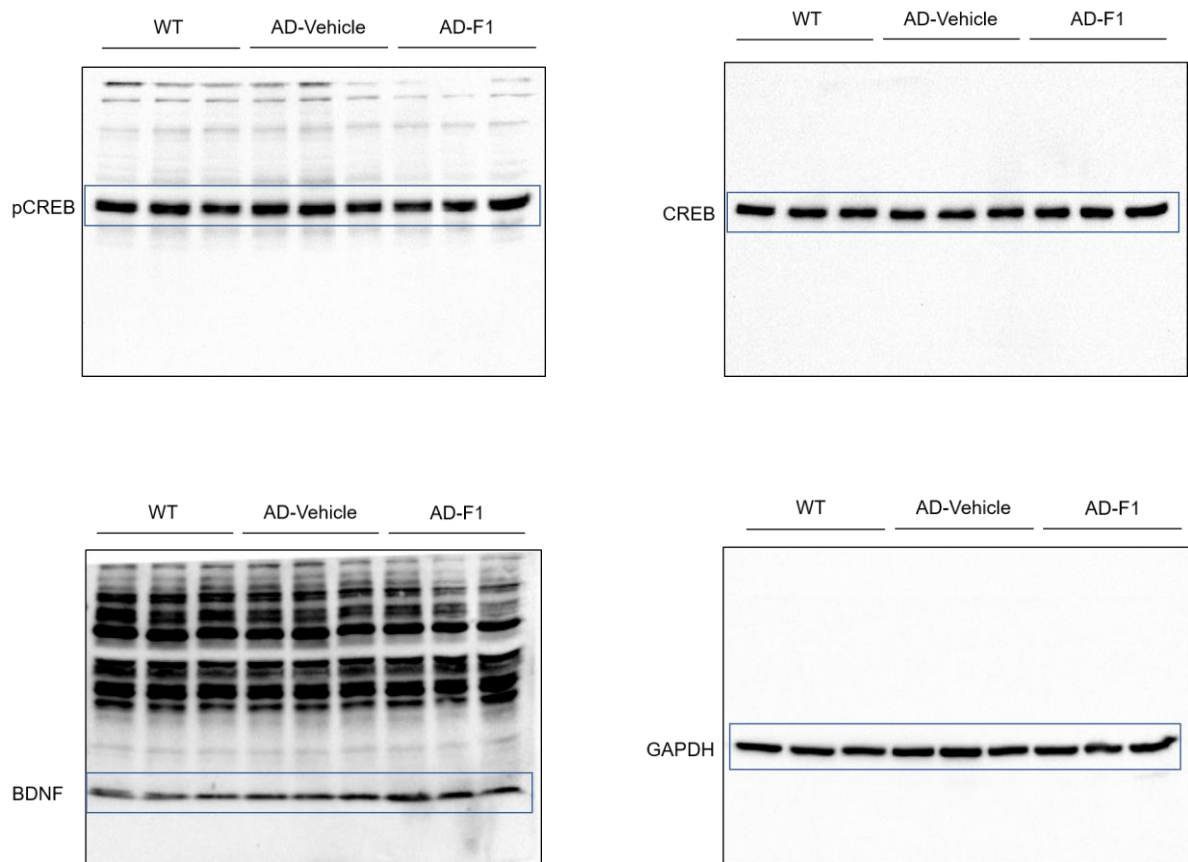

Supplement: Supplementary file 2 — Figure S2. Uncropped western blot images for cortex samples related with Fig. 3e. (PDF 132 kb) [file 13041_2019_495_MOESM2_ESM.pdf]
